# Solid-phase synthesis of peptides containing aminoadipic semialdehyde moiety and their cyclisations

**DOI:** 10.1038/s41598-018-28798-9

**Published:** 2018-07-11

**Authors:** Monika Kijewska, Mateusz Waliczek, Marta Cal, Łukasz Jaremko, Mariusz Jaremko, Maria Król, Marta Kołodziej, Marek Lisowski, Piotr Stefanowicz, Zbigniew Szewczuk

**Affiliations:** 1 Faculty of Chemistry, University of Wrocław, Joliot-Curie 14, 50-383 Wrocław, Poland; 2 King Abdullah University of Science and Technology (KAUST), Biological and Environmental Sciences & Engineering Division (BESE), Thuwal, 23955-6900 Saudi Arabia

## Abstract

Pathological levels of oxidative stress (OS) have been implicated in many diseases including diabetes mellitus, neurodegenerative diseases, inflammatory diseases, atherosclerosis, and cancer. Studies of oxidative stress are however complicated by the low concentration of oxidation products. To resolve this problem, we tested a new derivative of aminoadipic semialdehyde (Fmoc-Aea-OH) in the solid-phase synthesis of carbonylated peptides. We prepared a series of peptides with free and acetylated N-terminal amino groups using the Fmoc-Aea-OH reagent. LC-MS, ESI-MS, and MS/MS spectra confirmed the sequences of the modified peptides, although the LC-MS and ESI-MS spectra were dominated by signals corresponding to dehydration products. NMR studies of acetylated products revealed that the dominant product formed in this reaction contains a 1,2,3,4-tetrahydropyridine-2-carboxylic acid residue. Another side reaction in this system was the cleavage of the amide bond between the Aea residue and the amino acid moiety preceding it resulting in the formation of a side product with a six-membered ring at the N-terminus (2,3,4,5-tetrahydropyridine-2-carboxylic acid residue). We found that, depending on the peptide sequence, one of those side products is predominant. Our work suggests new methods for the solid-state synthesis of peptides containing unnatural amino acids.

## Introduction

Pathological levels of oxidative stress (OS) have been implicated in a plethora of diseases ranging from diabetes mellitus and neurodegenerative diseases to inflammatory diseases, atherosclerosis, and cancer^1–3^. Protein oxidation, which is a covalent modification of proteins induced either directly by reactive oxygen species (ROS) or indirectly by a reaction with secondary by-products of oxidative stress^4^, serves as a useful marker for assessing oxidative stress *in vivo*. Studies of oxidative stress are however complicated by the low concentration of oxidation products. There are many mechanisms of protein oxidation and, consequently, numerous types of oxidation products. One mechanism involves oxidative cleavage in either the protein backbone or the amino acid side chains^5–7^. Other moieties, especially Lys, Arg, Pro, and Thr^8^ incur formation of carbonyl groups (aldehydes and ketones) in the side chains. The second mechanism is based on the addition of lipid oxidation products, such as 4-hydroxy-2-nonenal, to proteins^2,9^. Finally, carbonyl groups in proteins can be generated by advanced glycation^10^.

Studies on the detection and quantification of post-translationaly modified peptides (e.g., carbonylated or glycated) require defined, analytically pure standards of modified peptides^11,12^. Recently, we performed the synthesis of carbonylated and glycated model peptides on a solid support using designed and synthesised building blocks^13,14^. These compounds were tested to confirm the identity of a glycated crystalline fragment found in biological material^11,14^. Our synthesized building block Fmoc-amino(2,5,5-trimetyhyl-1,3-dioxolan-2-yl)acetic acid (Fmoc-Atda-OH)^13^ allows the synthesis of peptides containing the D,L-Thr(O) residue, which may be used as models of oxidatively modified peptides that occur in biological systems and are related to many diseases^15,16^.

Here, we focus on another carbonyl modification naturally occurring in living organisms - aminoadipic semialdehyde. The biological importance of this modification was investigated when the properties of collagen were studied^17^. Moreover, α-amino adipic semialdehyde (α-AASA) accumulates in body fluids from patients with pyridoxine-dependent epilepsy because of mutations in antiquitin (ALDH7A1). It thus serves as a biomarker for this condition^18^. Specific cross-linked lysyl residues in collagen and elastin are converted to peptide-bound aminoadipic semialdehyde (allysine) by deamination and oxidation of the ε-carbon atom in lysine. The formation of allysine in collagen in bone is catalyzed by lysyl oxidase^19^. This enzyme is known to act only upon the short, non-triple helical ends of the collagen molecule. It is not possible to oxidize lysine peptides enzymatically because lysyl oxidase requires at least the intact tertiary structure^20^.

Two independent synthetic routes to prepare allysine di- and tripeptides and derivatives in solution were previously presented^21^. The first pathway uses conventional synthetic methods to yield the reduced form of allysine, 2-amino-6-hydroxyhexanoic acid. This compound is oxidized to the allysine derivative after introducing the protective groups at the amino- and carboxyterminus. In contrast, the second strategy introduces aldehyde at an early stage of synthesis. The function of the aldehyde is then protected by a dithioethylacetal derivative. In both cases, the only compound that can be isolated is a ring-closured product^21^. It is not possible to inhibit this undesired reaction completely by adding 2,4-dinitrophenylhydrazine or other aldehyde-trapping reagents. The formation of hydrazone is moderate, and the velocity of the ring-closure reaction increases. Finally, the basic conditions of the reduction facilitate the ring closure reaction, and the product is converted into the dehydropipecolic acid derivative^21^.

Here, we used a commercially available derivative of aminoadipic semialdehyde, Fmoc-Aea-OH (Fmoc-L-allysine ethylene acetal), containing the oxidative modification occurring in living organisms, in the solid-phase synthesis of carbonylated peptides. Our goal was to determine the optimal conditions for the routine synthesis of peptides containing carbonyl groups on a solid support. Further, we checked the reactivity of the aldehyde group in the peptide sequence to determine the influence of different amino acid residues on the formation of the desired carbonylated peptide. We found that the desired products were obtained in trace amounts. We used high-performance liquid chromatography coupled with a mass spectrometer with an electrospray ion source (LC-MS), tandem mass spectrometry (ESI-MS/MS), and nuclear magnetic resonance spectroscopy (NMR) to identify and characterise the main products. Our work suggests new methods for the solid-state synthesis of peptides containing unnatural amino acids.

## Results and Discussion

We tested the commercially available aminoadipic semialdehyde derivative Fmoc-Aea-OH (Fmoc-L-allysine ethylene acetal) as a reagent for the synthesis of carbonylated peptides. The peptides were prepared using a manual solid-phase technique based on the standard Fmoc synthetic strategy with TBTU as a coupling reagent. The reaction products were cleaved from the resin using trifluoroacetic acid/water (95:5, v/v). A series of synthetic model peptides (H-Ala-Aea-Ala-Phe-OH, Ac-Ala-Aea-Ala-Phe-OH, H-Gly-Aea-Gly-Ala-Phe-OH, Ac-Gly-Aea-Gly-Ala-Phe-OH, H-Ala-Glu-Gly-Aea-Gly-Ala-Phe-OH, Ac-Ala-Glu-Gly-Aea-Gly-Ala-Phe-OH, Ac-Gly-Aea-Lys-Gly-Gly-Gly-NH_2_, Ac-Gly-Aea-Ala-Ala-Ala-Ala-Ala-NH_2_, Ac-Gly-Aea-Asp-Gly-Arg-Thr-Leu-NH_2_, Ac-Glu-Aea-Asp-Gly-Arg-Thr-Leu-NH_2_, Ac-Lys-Aea-Asp-Gly-Arg-Thr-Leu-NH_2_) containing the Fmoc-Aea-OH building block was synthesized and purified as described in the Experimental Section. Data on all synthesized peptides and obtained side products are presented in Table S1 in Supporting Information. To make this model more realistic, we based the sequences of three peptides on a fragment from ubiquitin (a six amino acid peptide fragment [52–57]). The pentapeptide (H-Asp-Gly-Arg-Thr-Leu-OH) had been determined to be the shortest and most effective immunosuppressive fragment of ubiquitin^22,23^.

**Figure 1:**
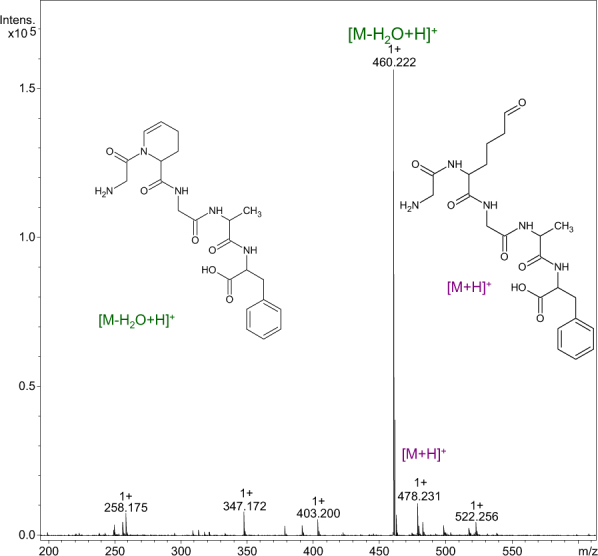
The ESI-MS spectrum of crude product H-Gly-Aea-Gly-Ala-Phe-OH.

**Figure 2:**
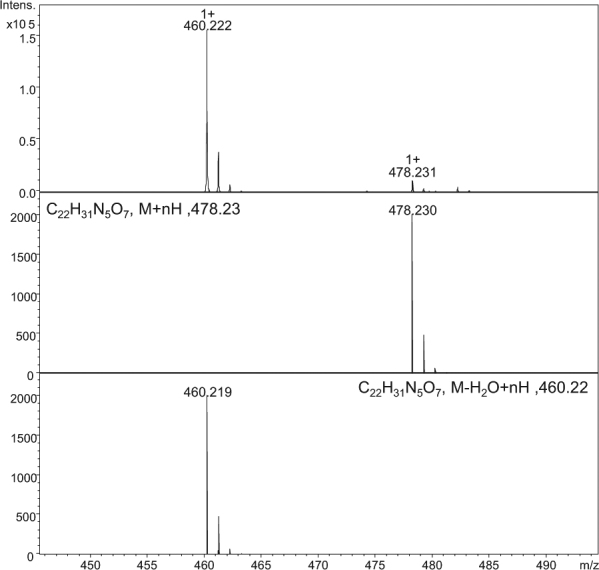
The ESI-MS spectrum of crude product H-Gly-Aea-Gly-Ala-Phe-OH and the simulated mass spectra of the desired *(m/z* 478.231) and dehydrated (*m/z* 460.222) products.

A representative ESI-MS spectrum of the crude product H-Gly-Aea-Gly-Ala-Phe-OH is presented in Fig. 1. The most abundant signal corresponds to the dehydration product of the target molecule. The peak of the desired product at *m/z* 478.23 is also observed but its intensity is relatively low. It was subjected to MS/MS analysis and the product structure was confirmed (Fig. S1). The simulated isotopic patterns for the desired product and the molecule obtained by its dehydration are presented in Fig. 2. A possible structure of the dehydrated product was proposed based on the fragmentation spectrum (Supporting Information, Fig. S2). The LC-MS analysis confirmed that the dehydrated product is not formed in the ion source. According to the literature, it is well known that under these conditions a Schiff base may be formed between the amino and carbonyl groups^24^, but based on our further investigations, we propose a more likely structure containing the six-membered ring.

**Figure 3:**
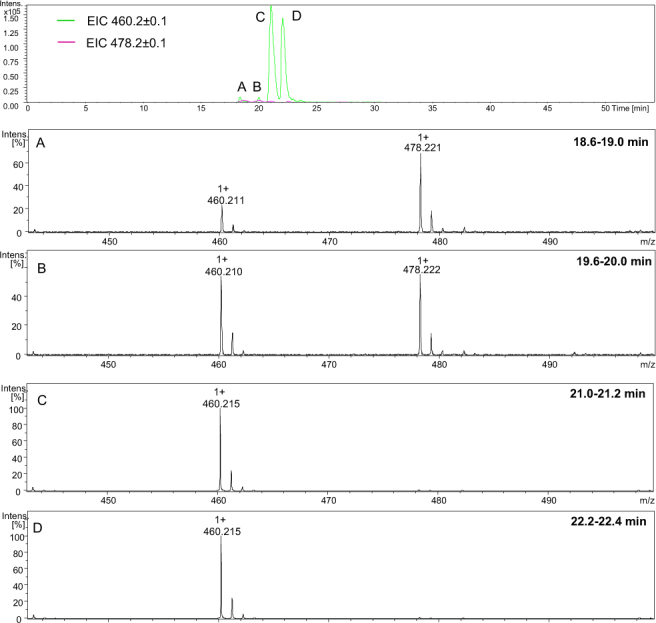
The LC-MS chromatogram of crude product H-Gly-Aea-Gly-Ala-Phe-OH (top); (**A**–**D)** ESI-MS spectra of peptides eluted at different retention times (see the experimental section for LC-MS experimental details).

The LC-MS analysis of the H-Gly-Aea-Gly-Ala-Phe-OH peptide is presented in Fig. 3. Extracted ion chromatograms for two main signals (*m/z* 460.211 and *m/z* 478.221) identified in the mass spectrum were generated. Two signals corresponding to the same molecular mass were observed. The ESI-MS spectra presented for signals A (Fig. 3, panel A and B) (Fig. 3, panel B) are the same and revealed the presence of both the desired product, containing a carbonyl group, and the dehydrated product formed in the ion source. Co-elution of the desired and dehydrated forms confirmed that these two peaks correspond to the same substance, which is not stable under the conditions of the ion source. Regarding peaks C and D: only dehydrated species were detected. This suggests that these dehydrated species are different than those observed in peaks A and B. Additionally, the double peaks were registered on a chromatogram (pairs A-B and C-D) with the same molecular masses, which suggests racemization at the α carbon.

**Figure 4:**
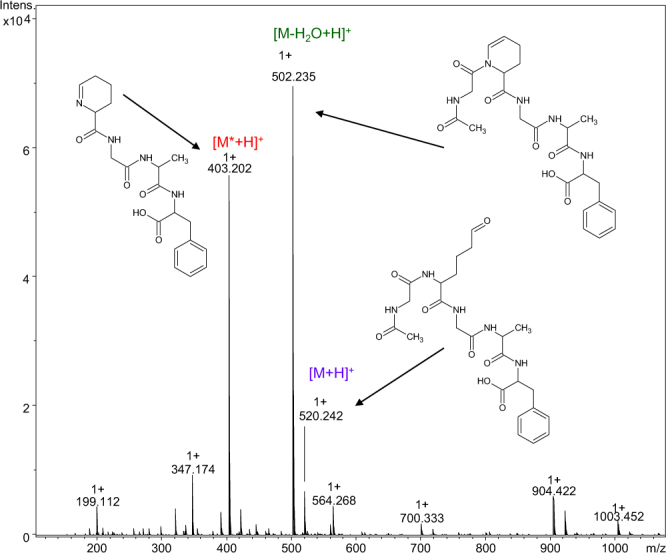
The ESI-MS spectrum of crude product Ac-Gly-Aea-Gly-Ala-Phe-OH.

**Figure 5:**
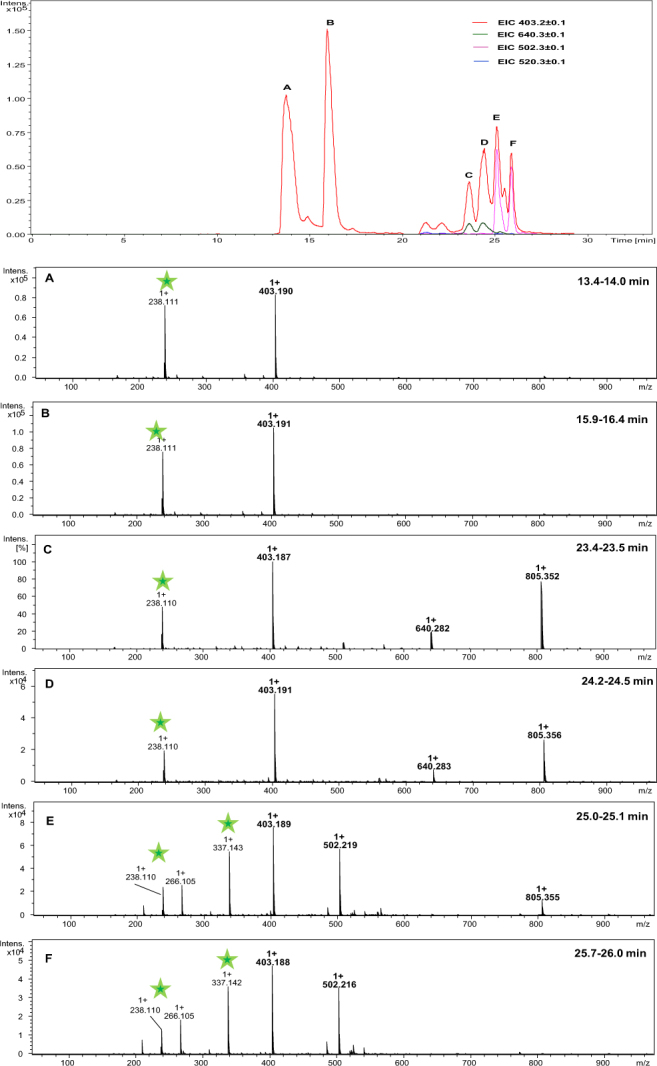
LC-MS of crude product Ac-Gly-Aea-Gly-Ala-Phe-OH; (**A**–**F**) ESI-MS spectra of peptides eluted at different retention times (green stars – fragmentation ions; the signal at *m/z* 805.35 is a non-covalent dimmer of 403.19 formed from the ion source) (see the LC-MS details in the experimental section).

Because the formation of a Schiff base requires a free amino group, our next step was to examine the acetylated peptide (Ac-Gly-Aea-Gly-Ala-Phe-OH). After cleavage of the product from the resin, the ESI-MS spectrum was measured (Fig. 4). Although acetylation excludes formation of the Schiff base, the dehydrated product ion (*m/z* 502.235) still appears as a predominant signal in the mass spectrum. We also found a signal corresponding to the desired product (*m/z* 520.242) but its intensity was significantly lower than that of the dehydrated product and the peak at *m/z* 403.202. The LC-MS analysis unambiguously confirmed that the dehydrated compound is not formed in the ion source. LC-MS analysis unambiguously showed that the peak at *m/z* 403.202 was registered for all six LC-MS signals (Fig. 5). The ESI-MS/MS studies (Fig. S3) revealed that it is due to the fragment ion that can be formed from the dehydrated compound (*m/z* 502.235). On the other hand, the product at *m/z* 403.202 corresponding to two main chromatographic peaks in Fig. 5(A,B) has a retention time that is different from the dehydration product, suggesting that the molecular mass of this product belongs to another side product. The presence of two chromatographic peaks with close retention times, intensities, and the same molecular masses may be the result of racemization.

**Figure 6:**
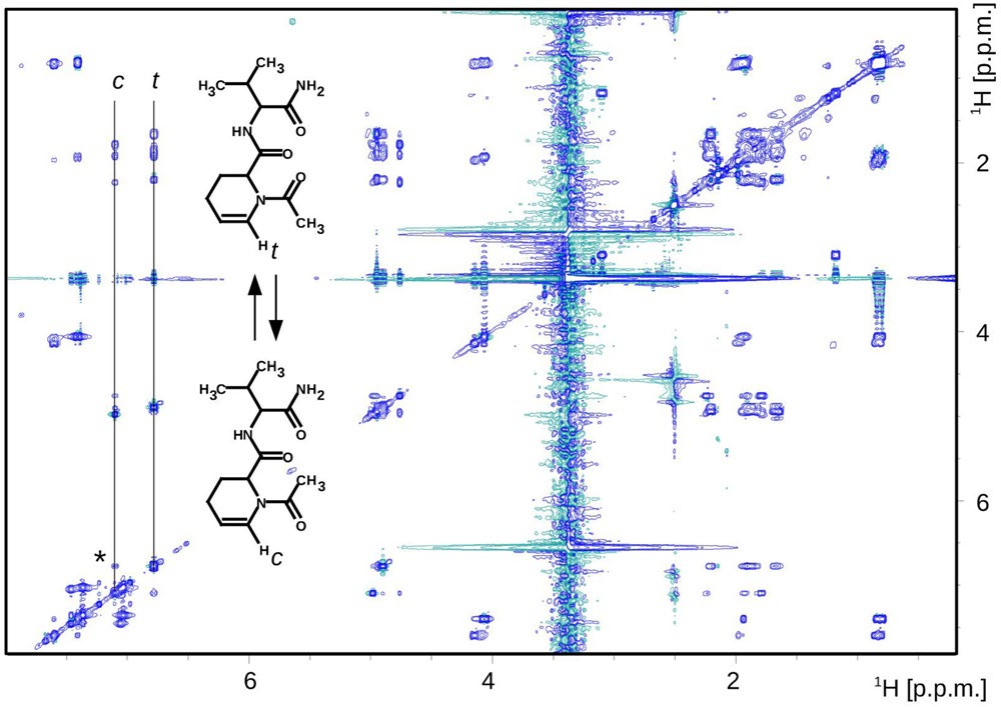
The 2D 1H-1H TOCSY (TOtal Correlated SpectroscopY) NMR spectrum recorded with an 80-ms mixing time of the dehydration product of Ac-Aea-Val-NH_2_.

**Figure 7:**
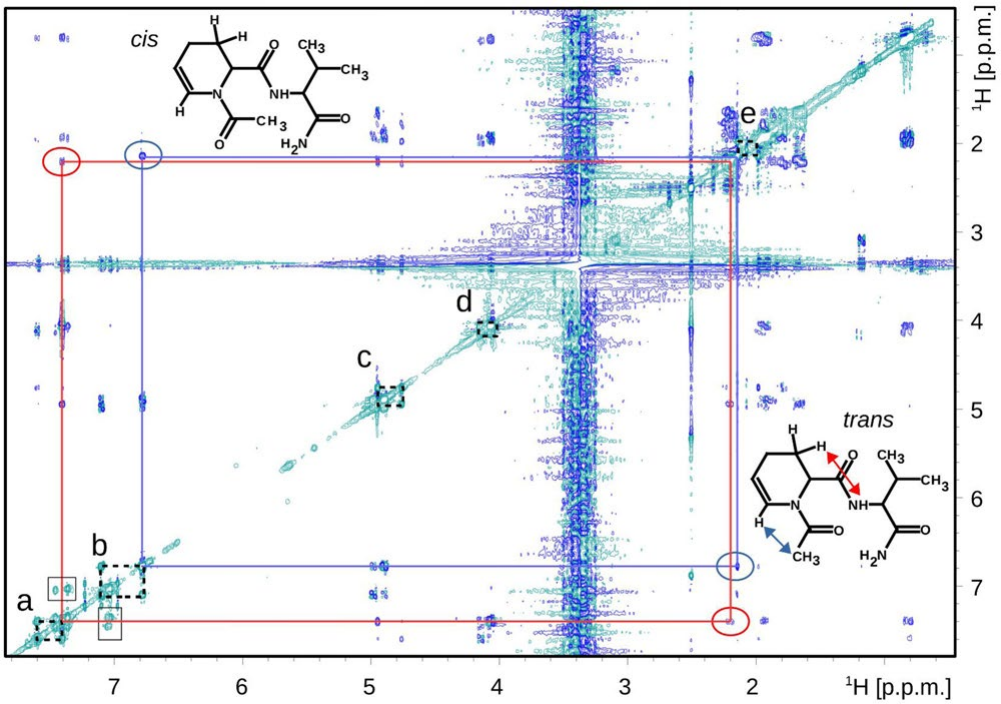
The 2D 1H-1H ROESY (Rotating frame Overhauser Effect SpectroscopY) NMR spectrum recorded with a 300-ms mixing time of the dehydration product of Ac-Aea-Val-NH_2_.

**Figure 8:**
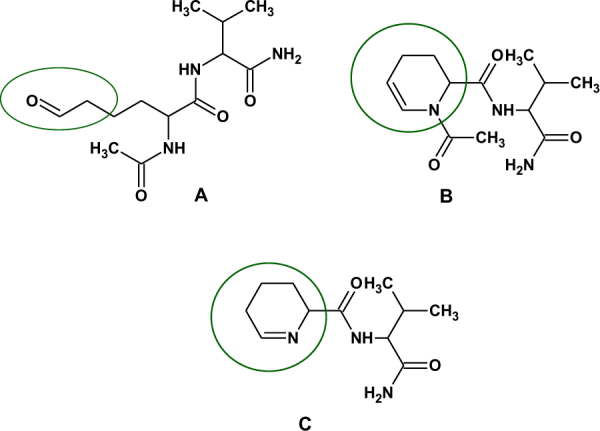
The structures of the desired (**A**) and actually obtained in synthesis (**B**,**C**) products.

To explain the formation of dehydrated peptides, we performed NMR studies on two model peptides, Ac-Aea-Val-NH_2_ and H-Aea-Val-NH_2_ (Figs 6, 7, and S4). Analysis of 2D NMR spectra of Ac-Aea-Val-NH_2_ revealed that Structure B (Fig. 8) is the main product obtained during the synthesis. The dehydrated compound exists in the equilibrium between two conformational isomers *cis* (c) and *trans* (t), around the >N-Ac bond. The chemical exchange peak between two vinyl protons is marked with a star (*) and the TOCSY- (TOtal Correlated SpectroscopY) type correlation strips from the distinguished protons (H_*c*_ and H_*t*_) of two coexisting isomers are marked with the solid horizontal lines. The *cis:trans* signal intensity ratio is about 1 to 3 (Fig. 6). In Fig. 7, we present a 2D ^1^H-^1^H ROESY (Rotating frame Overhauser Effect SpectroscopY) NMR spectrum recorded with a 300-ms mixing time of the dehydration product of Ac-Aea-Val-NH_2_. The rectangles marked with black dotted lines (a-e) indicate the additional exchange signals of the protons between the cis/trans isomers. The red and blue arrows indicate the through-space contacts (<5 Å). The contact marked in blue is specific to the trans isomer only. The contact indicated by the red arrow is also observed for the *cis* form although it is not shown in the figure for clarity. The corresponding signals in the ROESY correlation map are linked by solid red/blue lines and indicated by red/blue ellipses. The chemical formulas of the discussed isomers are depicted next to the characteristic correlation signals. The 2D ^1^H-^13^C HSQC (Heteronuclear Single Quantum Coherence) NMR spectrum of the isomeric mixture of the dehydration products of Ac-Aea-Val-NH_2_ can be seen in Supporting Information (Fig. S2). On the other hand, analysis of the 2D NMR spectra revealed that the main product formed during the synthesis of H-Aea-Val-NH_2_ corresponds to the Schiff base–type product with an N-terminal 2,3,4,5-tetrahydropyridine-2-carboxylic acid residue (Fig. 8C). The resonance assignments are given in the Experimental section.

**Figure 9:**
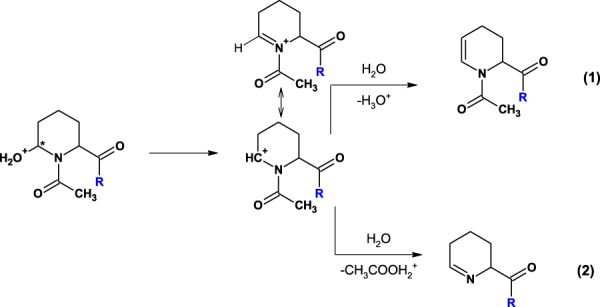
The proposed mechanism of cyclic side product formation (* - stereogenic center).

Besides the dehydrated product containing the N-acetylated 1,2,3,4-tetrahydropyridine-2-carboxylic acid residue (at *m/z* 502.235) in the acetylated model peptide, Ac-Gly-Aea-Gly-Ala-Phe-OH, another side product, containing the 2,3,4,5-tetrahydropyridine-2-carboxylic acid residue at *m/z* 403.202, was observed. Formation of this product is a result of peptide bond cleavage between Gly and Aea. Its structure is presented in Fig. 4. To rationalize the obtained data, we proposed a possible mechanism of the formation of cyclic products in this reaction. Under acidic conditions, the protonated carbonyl group is attacked by the nitrogen of the amide bond. Elimination of a water molecule produces the carbocation stabilized by the electron pair of the nitrogen atom. There are two possible paths of formation of different reaction products: (1) stabilization of the carbocation by elimination of a proton, and (2) formation of the product containing the N-terminal 2,3,4,5-tetrahydropyridine-2-carboxylic acid residue via stabilization of the carbocation by elimination of the acylium ion (under aqueous conditions as the protonated acid) (Fig. 9). A similar reaction was reported in the synthesis of (+)-epiquinamide. This reaction resulted in partial dimerization of the enamide product in the presence of toluenesulphonic acid. The reaction was however performed in neat trifluoroacetic acid, resulting in a fast and clean cyclization^25^.

**Figure 10:**
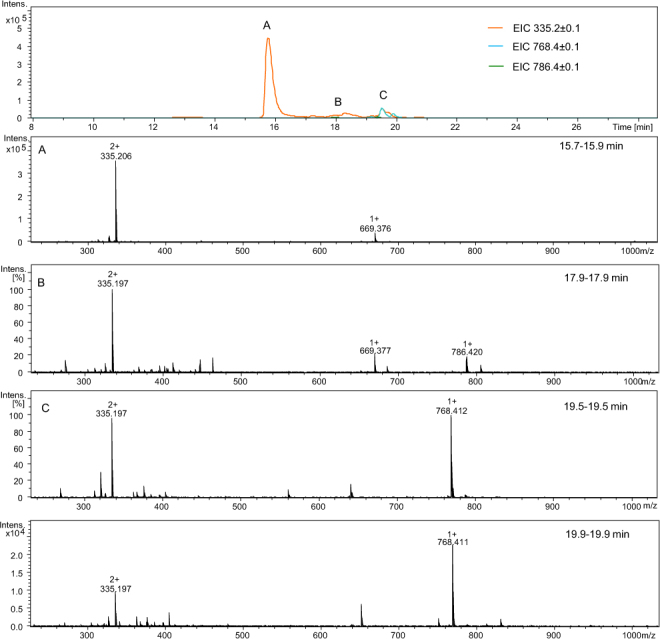
LC-MS of crude product Ac-Gly-Aea-Asp-Gly-Arg-Thr-Leu-NH_2_; (**A–D**) ESI-MS spectra of peptides eluted at different retention times (see the experimental section for LC-MS details).

**Figure 11:**
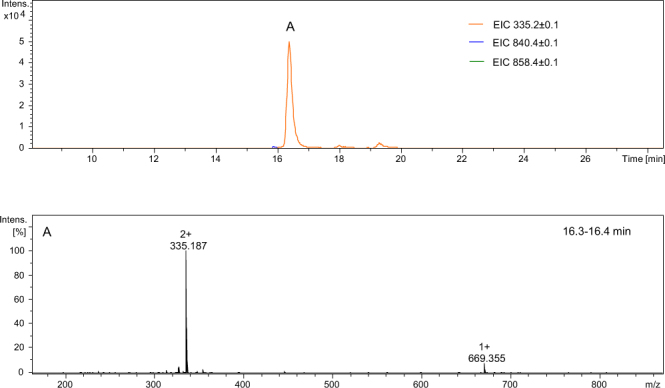
LC-MS of crude product Ac-Glu-Aea-Asp-Gly-Arg-Thr-Leu-NH_2_; (**A**) ESI-MS of the peptide eluted at 16.3–16.4 min (see the experimental section for LC-MS details).

**Figure 12:**
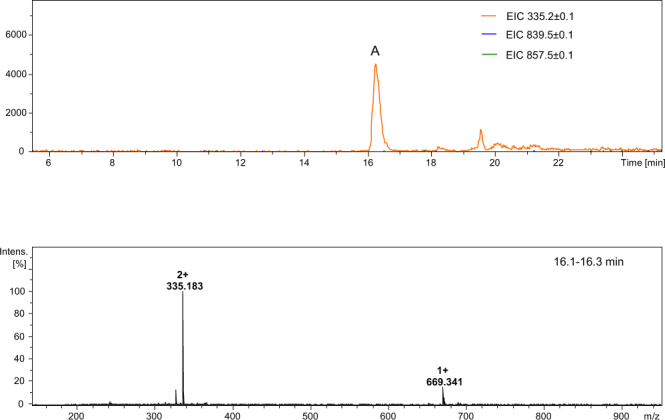
LC-MS of crude product Ac-Lys-Aea-Asp-Gly-Arg-Thr-Leu-NH_2_; (**A**) ESI-MS of the peptide eluted at 16.1–16.3 min (see the experimental section for LC-MS details).

We investigated the influence of two factors on the relative amount of both six-membered ring side products obtained during the synthesis. Five peptides (Ac-Gly-Aea-Lys-Gly-Gly-Gly-NH_2_, Ac-Gly-Aea-Ala-Ala-Ala-Ala-Ala-NH_2_, Ac-Gly-Aea-Asp-Gly-Arg-Thr-Leu-NH_2_, Ac-Glu-Aea-Asp-Gly-Arg-Thr-Leu-NH_2_, and Ac-Lys-Aea-Asp-Gly-Arg-Thr-Leu-NH_2_) were synthesized and subjected to ESI-MS and LC-MS analysis. We took into consideration the amino acid residues placed before and after the Aea moiety. In Ac-X-Aea-Asp-Gly-Arg-Thr-Leu-NH_2_, where X – Gly, Lys or Glu, the same side product dominates in the chromatogram (Figs 10–12**)**. These amino acid sequences favor disintegration of the amide bond between X and Aea and formation of 2,3,4,5-tetrahydropyridine-2-carboxylic acid at the N-terminus which may be a result of the presence of the aspartic acid moiety in the proximity of Aea. Dolz *et al*.^21^ noticed that polar groups seem to promote the ring closure. On the other hand, only in the peptide with X = Gly (Fig. 10) was a trace amount of the desired carbonylated product (*m/z* 786.455) observed. The ESI-MS spectra of all obtained peptides are presented in the Supplementary Materials (Figs S7, S6, and S7).

**Figure 13:**
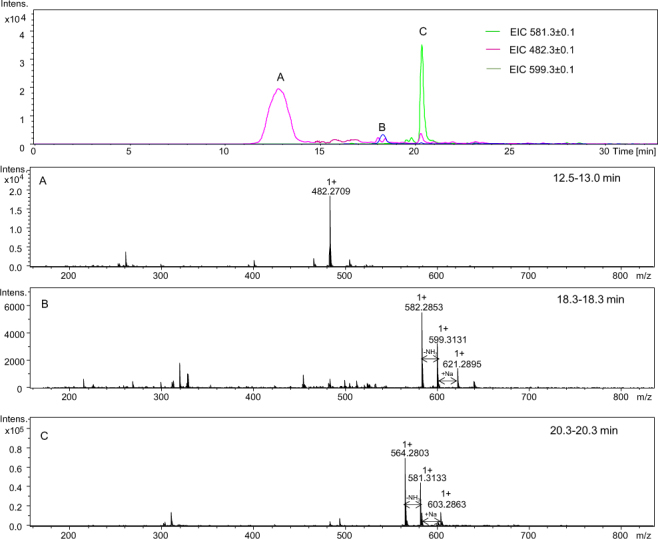
LC-MS of crude product Ac-Gly-Aea-Ala-Ala-Ala-Ala-Ala-NH_2_; (**A**–**C**) ESI-MS spectra of peptides eluted at different retention times (see the experimental section for LC-MS details).

**Figure 14:**
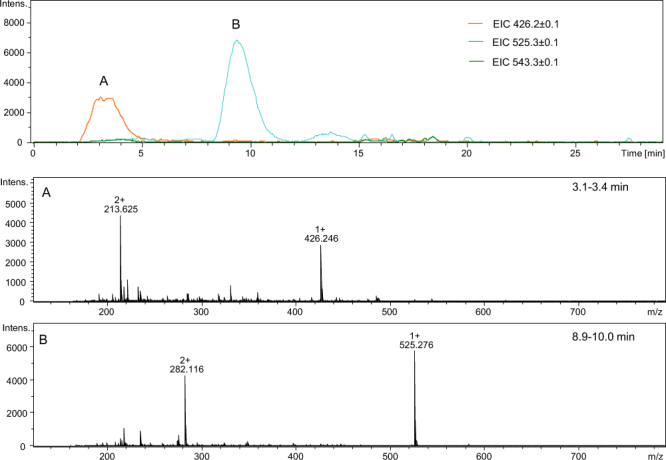
LC-MS of crude product Ac-Gly-Aea-Lys-Gly-Gly-Gly-NH_2_; (**A**) ESI-MS of the peptide eluted at 3.1–3.4 min; (**B**) ESI-MS of the peptide eluted at 8.9–10.0 min (see the experimental section for LC-MS details).

Next, we checked the influence of a side chain of the amino acid placed after the Aea residue on the yield of six-membered products because we noticed that a polar amino acid in this position may have a strong effect on the ring closure and cleavage amide of the bond. In the Ac-Gly-Aea-Ala-Ala-Ala-Ala-Ala-NH_2_ and Ac-Gly-Aea-Lys-Gly-Gly-Gly-NH_2_ examples, we placed Gly before the Aea residue and checked the influence of the polar and non-charged amino acid residues placed after the Aea. The obtained results are presented in Figs 13 and 14. In the peptides studied, cleavage of the amide bond between the Gly and Aea amino acid residues in both sequences occurs and corresponding peptides with the 2,3,4,5-tetrahydropyridine-2-carboxylic acid residue at the N-terminus are formed (Fig. 13 signal A at *m/z* 482.271 and Fig. 14 signal A at *m/z* 426.246). Furthermore, small amounts of the desired carbonylated peptides (Fig. 13 signal B at *m/z* 599.313) and the dehydrated products (Fig. 13 signal C at *m/z* 581.313 and Fig. 14 signal B at *m/z* 525.276) are also observed. In the chromatograms, the signals are broadened, which may suggest that racemization also occurred. Despite many attempts, we could not separate the racemic forms of the examined peptide sequences. The same phenomenon was observed in our previous investigations during the analysis of peptides comprising oxidized threonine, an unnatural amino acid. Previous LC-MS analysis confirmed racemization of carbonylated peptides^13^. Additionally, we performed fragmentation experiments for signals at *m/z* 482.26, 525.27, and 335.20 to confirm the structure of the obtained modified products **(**Figs S8, S9 and S10). A series of b and y ions allowed us to confirm the proposed structures.

## Conclusions

We investigated the use of an unnatural, commercially available derivative of aminoadipic semialdehyde (Fmoc-Aea-OH) as a building block in the solid-phase synthesis of modified peptides. The first assumption of the project was to obtain pure standards of carbonylated peptides and check the reactivity of aldehyde group. Using Fmoc-Aea-OH in a solid-phase synthesis resulted in the formation of two side reaction products. One of major products contained a 1,2,3,4-tetrahydropyridine-2-carboxylic acid residue formed by interaction of the protonated aldehyde group with the amide bond; the other contained the N-terminal 2,3,4,5-tetrahydropyridine-2-carboxylic acid residue resulting from cleavage of the peptide bond adjacent to the Aea residue. The ratio of these products depends on the peptide sequence. According to our best knowledge, this is the first report on the use of a derivative of aminoadipic semialdehyde in the solid-phase synthesis of peptides. Our results indicate that peptides comprising the aminoadipic semialdehyde derivative are reactive and under acidic conditions tend to undergo side reactions. In samples obtained from hydrolysis of the oxidized (carbonylated) proteins, these products may have appeared, but they were not taken into account in previous studies.

## Experimental

### Reagents

The derivatives of amino acids for peptide synthesis and the coupling reagent (TBTU) were purchased from NovaBiochem. The preloaded Wang (0.50–0.70 mmol/g) and Rink (0.68 mmol/g) resins were purchased from NovaBiochem. The solvents for peptide synthesis (analytical grade) were obtained from Riedel de Haën (DMF) and J. T. Baker (methanol). Other solvents used in this work were obtained from Aldrich.

### Peptide synthesis

The peptides were prepared by manual solid-support techniques using a standard Fmoc synthetic procedure^26^. The consecutive amino acid residues were coupled using TBTU (2-(1*H*-Benzotriazole-1-yl)-1,1,3,3-tetramethyluronium tetrafluoroborate) in DMF. The peptide was cleaved from the resin using trifluoracetic acid/water (95:5, v/v) for 2 h at room temperature. The peptides were isolated after evaporation of TFA in nitrogen stream and then the samples were lyophilized.

### Mass spectrometry measurements

Mass spectrometric measurements were performed on a quadrupole time-of-flight (micrOTOF-Q) instrument (Bruker, Germany) equipped with an electrospray ion source (ESI) ion funnel. Measurements were performed in the positive or negative ion mode and the apparatus was calibrated before each analysis with the Tunemix mixture (Bruker Daltonics) by a quadratic method. In the MS/MS experiments, the collision energy (5–20 eV) was optimized for the best fragmentation. An acetonitrile/water/formic acid (50:50:0.1) mixture or methanol were used as the solvents for recording the mass spectra. The potential between the spray needle and the orifice was set to 4.5 kV. In the MS/MS mode, the quadrupole was used to select the precursor ions, which were fragmented in the hexapole collision cell generating product ions that were subsequently mass analyzed by the orthogonal reflectron TOF mass analyzer. For the collision-induced dissociation (CID) MS/MS measurements, the voltage over the hexapole collision cell varied from 15 to 30 V and argon was used as a collision gas.

### LC-MS

The LC-MS analysis was performed on an Agilent 1200 HPLC system coupled to a micrOTOF-Q mass spectrometer (Bruker, Daltonics, Germany). Separation was carried out on an RP-Zorbax (50 × 2.1 mm, 3.5 µm) column with a gradient elution of 0–80% B in A (A, 0.1% HCOOH in water; B, 0.1% HCOOH in acetonitrile) at room temperature (flow rate: 0.1 mL/min) for 40 min.

### NMR

All NMR measurements for the Ac-Aea-Val-NH_2_ and H-Aea-Val-NH_2_ peptides were carried out on a 500 MHz spectrometer at 25 °C, at the peptide concentration of 4 mg mL^−1^, in 99% DMSO-*d*_6_. The complete proton resonance assignment of the Ac-Aea-Val-NH_2_ peptide spin systems was accomplished on the basis of 2D ^1^H–^1^H TOCSY (80 ms mixing times) and ^1^H–^1^H ROESY (300 ms) spectra.

**Ac-Aea-Val-NH**_**2**_ ***CIS*** **form**:

DMSO-*d*_6_,

^1^H resonances (ppm): 0.84 (3H, d, ^3^*J* = 6.9 Hz), 0.86 (3H, d, ^3^*J* = 6.9 Hz), 1.79 (1H, m), 1.83 (2H, m), 1.97 (3H, s), 1.99 (1H, m), 2.25 (1H, m), 4.15 (1H, dd, ^3^*J* = 6.5 Hz, ^3^*J* = 8.9 Hz), 4.76 (1H, broad), 4.98 (1H, m), 7.06 (1H, broad s), 7.12 (1H, m), 7.46 (1H, broad s), 7.60 (1H, d, ^3^*J* = 8.9 Hz);

^13^C resonances (ppm): 18,2, 18.6, 19.7, 21.5, 24.8, 30.8, 56.1, 57.9, 107.3, 123.7;

**Ac-Aea-Val-NH**_**2**_ ***TRANS*** **form**:

DMSO-*d*_6_,

^1^H resonances (ppm): 0.80 (3H, d, ^3^*J* = 6.4 Hz), 0.84 (3H, d, ^3^*J* = 6.4 Hz), 1.65 (1H, m), 1.91 (2H, m), 1.93 (1H, m), 2.15 (3H, s), 2.20 (1H, m), 4.07 (1H, dd, ^3^*J* = 6.4 Hz, ^3^*J* = 8.5 Hz), 4.90 (1H, m), 4.95 (1H, m), 7.04 (1H, broad s), 7.12 (1H, m), 7.36 (1H, broad s), 7.41 (1H, d, ^3^*J* = 8.5 Hz);

^13^C resonances (ppm): 18,2, 18.6, 19.8, 22.1, 24.1, 30.9, 52.5, 57.8, 106.6, 126.7;

**H-Aea-Val-NH**_**2**_ **Shiff-base**:

DMSO-*d*_6_,

^1^H resonances (ppm): 0.86 (3H, d, ^3^*J* = 6.7 Hz), 0.90 (3H, d, ^3^*J* = 6.7 Hz), 1.54 (1H, m), 1.56 (1H, m), 2.05 (3H, s), 2.09 (1H, m), 1.98 (1H, m), 3.72 (1H, m), 4.17 (1H, m), 6.40 (1H, broad s), 7.10 (1H, d), 7.49 (1H, d), 8.31 (1H, d, ^3^*J* = 9.2 Hz);

^13^C resonances (ppm): 18,3, 19.7, 23.0, 23.7, 26.9, 31.0, 53.3, 58.0, 133.1.

## Electronic supplementary material

Supplementary Information
