# Supplementary material for: Solid-phase synthesis of peptides containing aminoadipic semialdehyde moiety and their cyclisations
Source: Sci Rep. 2018 Jul 11;8:10462. doi: 10.1038/s41598-018-28798-9 (PMC6041278; doi:10.1038/s41598-018-28798-9)
Supplement: Supplementary file 1 — Supplementary Information [file 41598_2018_28798_MOESM1_ESM.docx]

**Supporting Information Scientific Reports**

**Solid-phase synthesis of peptides containing aminoadipic semialdehyde moiety and their cyclisations**

Monika Kijewska^1^, Mateusz Waliczek^1^, Marta Cal^1^, Łukasz Jaremko^2^, Mariusz Jaremko^2^ Maria Król^1^, Marta Kołodziej^1^, Marek Lisowski^1^, Piotr Stefanowicz^1^ and Zbigniew Szewczuk^1^

*^1^Faculty of Chemistry, University of Wrocław,* *Joliot-Curie 14, 50-383 Wrocław, Poland*

^2^ *King Abdullah University of Science and Technology (KAUST), Biological and Environmental Sciences &Engineering Division (BESE), Thuwal, 23955-6900, Saudi Arabia.*

**Corresponding author:** Monika Kijewska, Faculty of Chemistry, University of Wrocław, F. Joliot-Curie 14, 50-383 Wrocław, Poland, Fax: +48‑71‑3282348, Tel.: +48-71-3757250, E‑mail: [monika.kijewska@chem.uni.wroc.pl](mailto:monika.kijewska@chem.uni.wroc.pl)

Mariusz Jaremko, King Abdullah University of Science and Technology (KAUST), Biological and Environmental Sciences &Engineering Division (BESE), Thuwal, 23955-6900, Kingdom of Saudi Arabia, e-mail: [mariusz.jaremko@kaust.edu.sa](mailto:mariusz.jaremko@kaust.edu.sa)

**Fig. S1** ESI-MS/MS for desire carbonylated peptide H-Gly-Aea-Gly-Ala-Phe-OH
(*m/z* 478.23; collision energy: 10 eV)

**Fig. S2** ESI-MS/MS for dehydrated product of peptide H-Gly-Aea-Gly-Ala-Phe-OH
(*m/z* 460.22; collision energy: 15 eV)

**Fig. S3** ESI-MS/MS for dehydrated product of peptide Ac-Gly-Aea-Gly-Ala-Phe-OH
(*m/z* 502.23; collision energy: 15 eV)


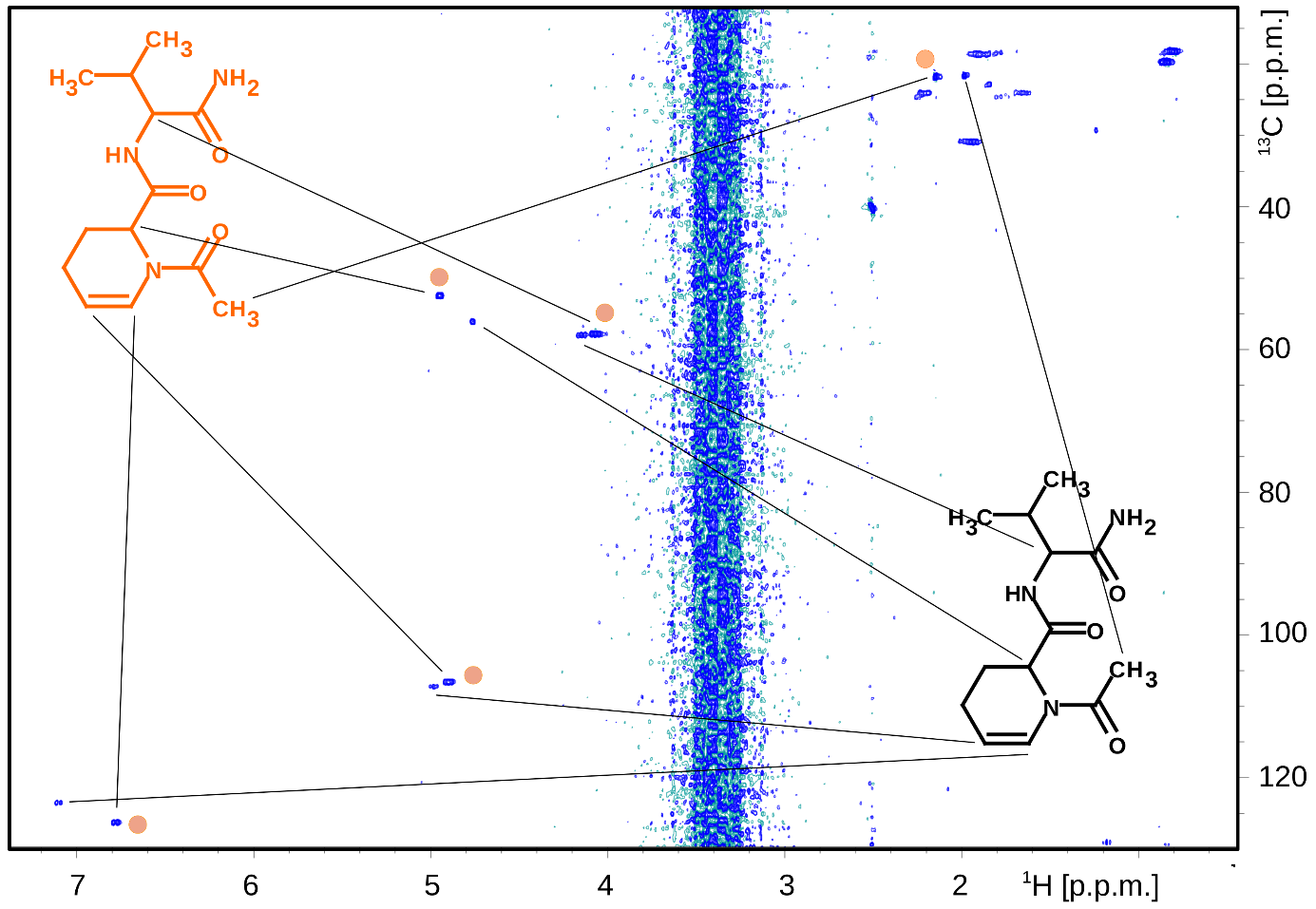


**Fig. S4** The 2D NMR spectrum of ^1^H-^13^C HSQC (Heteronuclear Single Quantum Coherence) of the isomeric mixture of the dehydration product of Ac-Aea-Val-NH_2_ peptide. The *trans* form is marked orange and corresponding ^1^H/^13^C correlations are assigned by solid lines and the *cis* form is marked black.

**Fig. S5** ESI-MS for crude product of peptide Ac-Glu-Aea-Asp-Gly-Arg-Thr-Leu-NH_2_

**Fig. S6** ESI-MS for crude product of peptide Ac-Lys-Aea-Asp-Gly-Arg-Thr-Leu-NH_2_

**Fig. S7** ESI-MS for crude product of peptide Ac-Gly-Aea-Asp-Gly-Arg-Thr-Leu-NH_2_

**Fig. S8** ESI-MS/MS for six membered ring side product of peptide Ac-Gly-Aea-Ala-Ala-Ala-Ala-Ala-NH_2_ (*m/z* 482.26; collision energy: 10 eV)

**Fig. S9** ESI-MS/MS for dehydrated product of peptide Ac-Gly-Aea-Lys-Gly-Gly-Gly-NH_2_ (*m/z* 525.27; collision energy: 15 eV)

**Fig. S10** ESI-MS/MS for six membered ring side product of peptide Ac-Gly-Aea-Asp-Gly-Arg-Thr-Leu-NH_2_ (*m/z* 335.2; collision energy: 8 eV)

**Table S1.** MS data for all synthesized modified peptides and their side products.

**For acetylated compounds**

| **Compound** |  | **mass calc.** | **mass found** |
| --- | --- | --- | --- |
| Ac-Ala-Aea-Ala-Phe-OH | [M+H]^+^ | 477.234 (1+) | 477.229 (1+) |
|  | [M-H_2_O+H]^+^ | 459.224 (1+) | 459.220 (1+) |
|  | [M*+H]^+^ | 346.176 (1+) | 346.176 (1+) |
| Ac-Gly-Aea-Gly-Ala-Phe-OH | [M+H]^+^ | 520.240 (1+) | 520.243 (1+) |
|  | [M-H_2_O+H]^+^ | 502.230 (1+) | 502.219 (1+) |
|  | [M*+H]^+^ | 403.198 (1+) | 403.202 (1+) |
| Ac-Ala-Glu-Gly-Aea-Gly-Ala-Phe-OH | [M+H]^+^ | 720.320 (1+) | 720.320 (1+) |
|  | [M-H_2_O+H]^+^ | 702.309 (1+) | 702.312 (1+) |
|  | [M*+H]^+^ | 403.198 (1+) | 403.206 (1+) |
| Ac-Gly-Aea-Lys-Gly-Gly-Gly-NH_2_ | [M+H]^+^ | 543.289 (1+) | NF |
|  | [M-H_2_O+H]^+^ | 525.278 (1+) | 525.276 (1+) |
|  | [M*+H]^+^ | 213.626 (2+) | 213.625 (2+) |
| Ac-Gly-Aea-Ala-Ala-Ala-Ala-Ala-NH_2_ | [M+H]^+^ | 599.315 (1+) | 599.313 (1+) |
|  | [M-H_2_O+H]^+^ | 581.304 (1+) | 581.313 (1+) |
|  | [M*+H]^+^ | 482.272 (1+) | 482.271 (1+) |
| Ac-Gly-Aea-Asp-Gly-Arg-Thr-Leu-NH_2_ | [M+H]^+^ | 786.410 (1+) | 786.428 (1+) |
|  | [M-H_2_O+H]^+^ | 768.401 (1+) | 768.419 (1+) |
|  | [M*+H]^+^ | 335.187 (2+) | 335.210 (2+) |
| Ac-Glu-Aea-Asp-Gly-Arg-Thr-Leu-NH_2_ | [M+H]^+^ | 858.432 (1+) | NF |
|  | [M-H_2_O+H]^+^ | 840.421 (1+) | 840.469 (1+) |
|  | [M*+H]^+^ | 335.187 (2+) | 335.207 (2+) |
| Ac-Lys-Aea-Asp-Gly-Arg-Thr-Leu-NH_2_ | [M+H]^+^ | 857.484 (1+) | NF |
|  | [M-H_2_O+H]^+^ | 420.243 (2+) | 420.258 (2+) |
|  | [M*+H]^+^ | 335.187 (2+) | 335.200 (2+) |

NF – not found

**For free N-terminal group compounds**

| **Compound** |  | **mass calc.** | **mass found** |
| --- | --- | --- | --- |
| H-Ala-Aea-Ala-Phe-OH | [M+H]^+^ | 435.224 (1+) | 435.220 (1+) |
|  | [M-H_2_O+H]^+^ | 417.213 (1+) | 417.209 (1+) |
| H-Gly-Aea-Gly-Ala-Phe-OH | [M+H]^+^ | 478.230 (1+) | 478.222 (1+) |
|  | [M-H_2_O+H]^+^ | 460.219 (1+) | 460.211 (1+) |
| H-Ala-Glu-Gly-Aea-Gly-Ala-Phe-OH | [M+H]^+^ | 678.309 (1+) | 678.310 (1+) |
|  | [M-H_2_O+H]^+^ | 660.299 (1+) | 660.302 (1+) |
